# Oxidative cell death in the central nervous system: mechanisms and therapeutic strategies

**DOI:** 10.3389/fcell.2025.1562344

**Published:** 2025-04-30

**Authors:** Nan Liu, Ya Liu, Yingzhao Wang, Chunsheng Feng, Meihua Piao, Ming Liu

**Affiliations:** ^1^ Department of Anesthesiology, the First Hospital of Jilin University, Changchun, Jilin, China; ^2^ Department of Nutrition and Food Safety, School of Public Health, Jilin University, Changchun, Jilin, China; ^3^ Department of Neurology, Qianwei Hospital of Jilin Province, Changchun, Jilin, China; ^4^ Department of Neurosurgery, Qianwei Hospital of Jilin Province, Changchun, Jilin, China

**Keywords:** oxidative stress, central nervous system, neurodegenerative diseases, antioxidant therapy, neuroprotection

## Abstract

Oxidative cell death is caused by an overproduction of reactive oxygen species and an imbalance in the antioxidant defense system, leading to neuronal dysfunction and death. The harm of oxidative stress in the central nervous system (CNS) is extensive and complex, involving a variety of molecular and cellular level changes that may lead to a variety of acute and chronic brain pathologies, such as stroke, traumatic brain injury, or neurodegenerative diseases and psychological disorders. This review provides an in-depth look at the mechanisms of oxidative cell death in the central nervous system diseases. In addition, the review evaluated existing treatment strategies, including antioxidant therapy, gene therapy, and pharmacological interventions targeting specific signaling pathways, all aimed at alleviating oxidative stress and protecting nerve cells. We also discuss current advances and challenges in clinical trials, and suggest new directions for future research, including biomarker discovery, identification of potential drug targets, and exploration of new therapeutic techniques, with a view to providing more effective strategies for the treatment of CNS diseases.

## 1 Introduction

The CNS, comprising the brain and spinal cord, serves as the nucleus of the nervous system and is crucial for processing information and controlling emotions, thoughts, actions, and feelings. It sustains life and regulates key functions, including heartbeat and breathing, enabling us to perceive the world, learn, remember, make decisions, and communicate effectively with others (Bassi et al., 2024). In addition, the central nervous system maintains the body’s internal balance through the endocrine and autonomic nervous systems, helping us adapt to and respond to our environment (Mota and Madden, 2024). Given the critical role of the CNS in maintaining our health and daily lives, it is essential to understand and mitigate the factors that can compromise its integrity.

Oxidative stress, a state where the oxidative and antioxidant systems are out of balance, leading to potential cellular damage. This imbalance is usually caused by an excessive accumulation of ROS, which are oxygen-containing molecules produced during cellular metabolism that are highly reactive (Lee et al., 2021). Under normal physiological conditions, ROS plays an important role in cell signaling, cell growth, differentiation and immune response. However, when the production of ROS exceeds the capacity of the cell’s antioxidant defense system, oxidative stress occurs, which may lead to DNA damage, protein oxidation, lipid peroxidation, and ultimately cell death (Wang et al., 2024e). In the context of the CNS, oxidative stress can induce oxidative cell death in neurons through several mechanisms. For example, it can cause accumulation of misfolded proteins, mitochondrial dysfunction, leading to the release of pro-apoptotic factors and subsequent cell death (Nguyen et al., 2024). Oxidative cell death is particularly significant due to the high metabolic activity and limited regenerative capacity of neurons. This type of cell death can lead to a variety of pathological changes, including neurodegenerative diseases such as Alzheimer’s disease (AD), Parkinson’s diseases (PD), cerebrovascular diseases such as stroke, psychiatric disorders such as depression and schizophrenia, neurodevelopmental disorders such as autism spectrum disorders, nerve damage and neuralgia, and brain tumors (Liu et al., 2023; Liu et al., 2024 B.; Fujikawa et al., 2024; Hussain et al., 2024; Miao et al., 2024; Wang et al., 2024d; Zhang et al., 2024b; de Souza et al., 2025). Therefore, reducing oxidative stress through antioxidant interventions and lifestyle improvements may help prevent or slow the onset and progression of these diseases. This review aims to explore the mechanism of oxidative cell death in CNS and its association with various neurological diseases, evaluate the therapeutic potential of oxidative cell death, provide new directions for the treatment of CNS diseases, and comprehensively analyze existing therapeutic strategies and clinical trial progress, so as to provide scientific basis and inspiration for future research and potential new therapy development.

## 2 Mechanism of oxidative cell death

### 2.1 Production of ROS

ROS, which include superoxide anion (O_2_−), hydrogen peroxide (H_2_O_2_), hydroxyl radical (OH−), ozone (O_3_), and singlet oxygen (^1^O_2_), are highly reactive oxygen-containing substances with unpaired electrons produced during cellular metabolism. The primary sources of ROS within the cell are mitochondria, particularly complex I and Complex III of the mitochondrial respiratory chain, where electrons are sometimes directly transferred to oxygen molecules to form superoxide anion (O_2_−), which can then be converted to hydrogen peroxide (H_2_O_2_) (Lennicke and Cochemé, 2021). Additionally, the NADPH oxidase family (NOX family) on the plasma membrane of cells is a significant generator of ROS, transferring electrons from NADPH to oxygen molecules to produce superoxide anions that form H2O2, with the NOX family playing a crucial role in immune response and signal transduction, with different expressions in various tissues (Magnani and Mattevi, 2019). The endoplasmic reticulum (ER) also contributes to ROS production, especially through protein disulfide isomerase during protein oxidation, primarily generating hydrogen peroxide (Teng and Zhao, 2024). Peroxisomes produce ROS, including hydrogen peroxide, through the oxidation of fatty acids and other substances (Bahraini et al., 2024). Cytoplasmic enzymes, such as xanthine oxidase, cyclooxygenase, cytochrome P450 enzyme, and lipoxygenase, are further sources of ROS(Aftab et al., 2024; García-García et al., 2024; Liu R. et al., 2024; Zhu and Liang, 2024). The production of ROS is regulated by various factors, including environmental influences like radiation, hyperbaric oxygen, cigarette smoke, and air pollution, metal ions such as lead, chromium, and vanadium, drugs including anticancer medications and antibiotics, metabolic conditions like hyperglycemia, inflammatory factors (He Y. et al., 2024; Kurniawan et al., 2024; Peña et al., 2024; Yao et al., 2024). At the physiological level, ROS can be used as signaling molecules to participate in cell signaling, regulating cell growth, differentiation and survival (Yang Y. et al., 2024). However, excessive ROS can cause oxidative damage to cell components, which is associated with the occurrence and development of various diseases, such as cancer, diabetes, cardiovascular disease and CNS diseases (Cheung et al., 2024; Dai et al., 2024; Li Q. et al., 2024; Liu et al., 2024b).

### 2.2 Effects of ROS on cells

An imbalance in cellular redox balance due to excessive ROS production affects a range of redox-sensitive signaling molecules, thereby influencing cell survival and death. The influence of ROS on cells is multifaceted, involving key physiological and pathological processes such as cell signaling, metabolic regulation and cell death (Huai et al., 2024). As a by-product of cell metabolism, ROS is produced during normal aerobic metabolism and plays an important signaling role within cells, capable of reversibly oxidizing key reduction-oxidation (REDOX) sensitive cysteine residues on target proteins, and these oxidative post-translational modifications (PTM) can control the biological activity of many enzymes and transcription factors (TFs) (Hua et al., 2024; Yin et al., 2024). ROS also regulates protein synthesis through oxidative modification of cysteine, affecting physiological processes such as cell growth and proliferation. ROS can disrupt cellular homeostasis by impairing protein homeostasis. Excessive ROS can cause protein misfolding, leading to the formation of protein aggregates. This accumulation of misfolded proteins can overwhelm the cellular quality control systems, ultimately resulting in cell death (Pathak et al., 2024). ROS also affects DNA damage repair, activates the DNA damage response pathway, and then activates the cell death signaling pathway (Zhang H. et al., 2024). In addition, ROS also can cause mitochondrial damage, increase the permeability of mitochondrial intima, release cytochrome C and other proteins, activate caspase, and finally induce cell apoptosis (Huang Q. et al., 2024). Excessive ROS can cause cell necrosis and induce autophagy in some specific conditions, which has dual effects on cells themselves (Bai et al., 2024). Finally, the ROS production and clearance system actively maintains the intracellular REDOX state, mediates REDOX signals, and regulates cell functions (Yang H. et al., 2024). Multiple ROS production and clearance systems, such as superoxide dismutase (SOD), glutathione peroxidase (GPX), glutathione S-transferase (GST), can clear ROS and maintain intracellular REDOX balance (Babu et al., 2024). To sum up, the effects of ROS on cells are complex and multidimensional, and they play a key role in cell physiology and pathology, including cell signaling, metabolic regulation, cell death and other mechanisms.

## 3 Characteristics of oxidative stress in CNS

CNS exhibits unique characteristics in response to oxidative stress, including its high metabolic activity, which results in the high production of ROS (Cui Y. et al., 2024). The abundant polyunsaturated fatty acids in nerve cell membranes make them especially vulnerable to ROS attacks, leading to lipid peroxidation. Compared to other cells in the body, nerve cells have a relatively weak antioxidant defense system and limited regenerative capacity, making them more sensitive to oxidative damage (Policastro et al., 2024). Higher levels of iron in the brain increase the risk of oxidative stress, and mitochondrial dysfunction further intensifies ROS production (Jia et al., 2023). DNA damage can lead to genetic mutations associated with the development of neurodegenerative diseases (Pranty et al., 2024). Abnormal autophagy may lead to an imbalance in the intracellular environment, and neurotransmitter imbalances may affect the normal function of the nervous system (Moon et al., 2024). The blood-brain barrier (BBB) is vital for maintaining CNS homeostasis by regulating the passage of substances between the bloodstream and the brain. However, in neuroinflammatory conditions, BBB integrity can be compromised, increasing permeability and allowing harmful substances such as pro-inflammatory cytokines and free radicals to enter the CNS, thereby exacerbating oxidative stress and neuronal damage. Oxidative stress can impair BBB function by damaging endothelial cells, disrupting tight junction proteins, and upregulating matrix metalloproteinases, further increasing permeability (Zandona et al., 2025). This creates a vicious cycle that can lead to neuroinflammation, neuronal dysfunction, and neurodegeneration. All of these factors combine to make the CNS particularly vulnerable to oxidative stress, which can lead to cell dysfunction and even death, and is closely associated with the development of multiple CNS diseases. Future research should focus on elucidating the molecular mechanisms of this interaction and exploring novel therapeutic agents targeting those pathways.

## 4 Oxidative stress and neurological diseases

**FIGURE 1:**
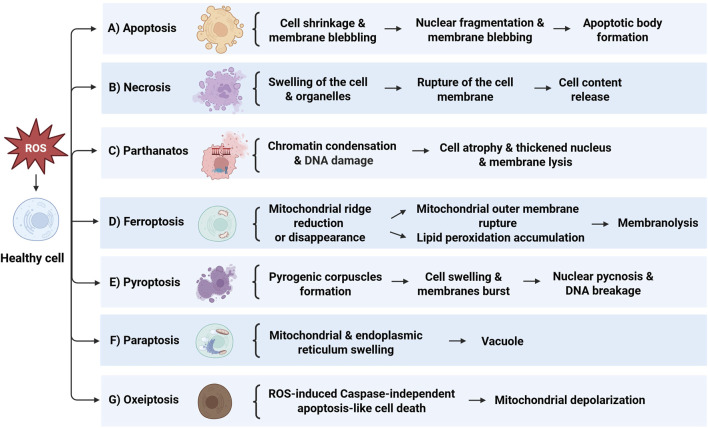
Overview of the mechanisms of cell death induced by ROS. **(A)** Apoptosis: Initiated by cell shrinkage and membrane blebbing, leading to nuclear fragmentation and apoptotic body formation. **(B)** Necrosis: Marked by swelling of the cell and organelles, culminating in rupture of the cell membrane and release of cell contents. **(C)** Parthanatos: Involves chromatin condensation and DNA damage, resulting in cell atrophy, thickened nucleus, and membrane lysis. **(D)** Ferroptosis: Characterized by mitochondrial ridge reduction or disappearance, mitochondrial outer membrane rupture, and lipid peroxidation accumulation, leading to membranolysis. **(E)** Pyroptosis: Features pyrogenic corpuscles formation, cell swelling, and membranes burst, followed by nuclear pyconosis and DNA breakage. **(F)** Paraptosis: Shown by mitochondrial and endoplasmic reticulum swelling, resulting in vacuole formation. **(G)** Oxeiptosis: Represents ROS-induced caspase-independent apoptosis-like cell death, associated with mitochondrial depolarization.

The cellular response to oxidative damage can take on various forms of cell death. This includes apoptosis, necrosis, and newly recognized forms like parthanatos, ferroptosis, pyroptosis, paraptosis and oxeiptosis, each with their own distinct molecular mechanisms and implications. These processes are extensively discussed in later sections of this review (Figure 1). The effects of oxidative damage on CNS cells vary across neurons, astrocytes, microglia, and oligodendrocytes. Neurons are highly susceptible to oxidative stress due to their high metabolic rate and limited regenerative capacity, leading to apoptosis *via* mitochondrial dysfunction and cytochrome C release (de Souza et al., 2025). Astrocytes are more resilient, producing antioxidants and neuroprotective factors, but can die under severe stress, contributing to neuroinflammation (Zufferey et al., 2025). Microglia, the CNS immune cells, activate inflammatory pathways in response to oxidative stress, which can exacerbate damage if chronically activated (Fujikawa et al., 2024). Oligodendrocytes, responsible for myelin production, are vulnerable to oxidative stress due to their high lipid content, and ROS-induced damage can cause demyelination and cell death, contributing to diseases like multiple sclerosis (van Veggel et al., 2025). Oxidative stress and neuroinflammation significantly contribute to the pathophysiology of psychiatric disorders. Elevated inflammatory cytokines like IL-6 and TNF-α, along with increased ROS production, are often observed in conditions such as depression and schizophrenia. These factors can activate microglia, leading to chronic inflammation, and cause oxidative damage to lipids, proteins, and DNA, impairing mitochondrial function and neurotransmitter regulation (Fujikawa et al., 2024; Liu B. et al., 2024). Recognizing the nuances of how oxidative stress induces cell death is crucial for developing targeted therapeutic strategies to combat conditions associated with excessive ROS production or impaired antioxidant defenses.

### 4.1 Apoptosis

Oxidative stress-induced apoptosis is a multifaceted process that engages various signaling pathways and molecular mechanisms. The accumulation of ROS can initiate apoptosis through several key mechanisms: Firstly, ROS can damage the mitochondrial membrane, leading to a decrease in mitochondrial membrane potential, which in turn promotes the release of proteins like cytochrome C (Liu et al., 2024c). These proteins then interact with apoptosis-related factors such as apoptotic protease activating factor-1 to form the apoptosome, activating the caspase cascade and ultimately resulting in apoptosis (Tang et al., 2024). Secondly, ROS can cause direct damage to DNA molecules; if the DNA repair mechanisms fail to address these lesions promptly, cells may trigger apoptosis through proteins like p53 (Huang H. et al., 2024). Additionally, ROS influence the expression of proteins associated with apoptosis, such as downregulating B-cell lymphoma 2 (Bcl-2) and upregulating Bax, which enhances mitochondrial permeability and initiates apoptosis (Hao et al., 2024). The production of ROS can also activate various signal transduction pathways, including MAPK, JNK, and ERK, which then activate transcription factors like nuclear factor κB (NF-κB), impacting cell survival and death (Lin et al., 2024). Oxidative stress induced by ROS may also lead to ER stress, activating apoptotic pathways related to ER stress, such as the Protein Kinase R-like Endoplasmic Reticulum Kinase (PERK)- Eukaryotic Initiation Factor 2α (eIF2α)-Activating Transcription Factor 4 (ATF4) pathway, which increases the expression of genes involved in apoptosis (Liu and Zhang, 2020). Moreover, the accumulation of ROS can activate autophagy, the cellular process of self-digestion to degrade and recycle damaged components, and excessive autophagy can also result in cell death.

**FIGURE 2:**
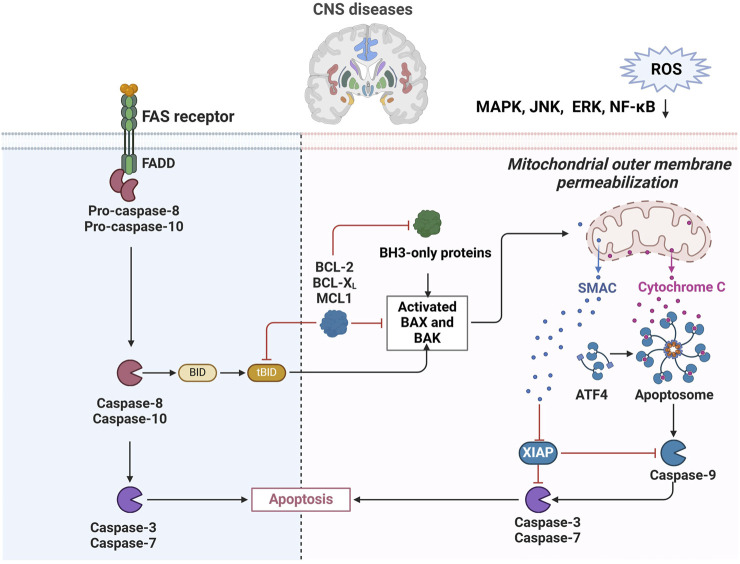
Regulation of apoptosis in CNS diseases. ROS-induced mitochondrial damage leading to cytochrome C release, activating caspase-9 and initiating apoptosis. The extrinsic pathway, activated by Fas receptor and FADD, involves caspase-8 and -10 to promote cell death. The diagram highlights how ROS affect signaling pathways like MAPK and NF-kB, influencing apoptosis in CNS diseases.

Apoptosis plays a key role in CNS diseases, which not only regulates the life and death of neural precursor cells, differentiated neurons and glial cells through the balance of Bcl-2 family proteins during neural development, but also shapes the neural structure. Moreover, abnormal neuronal apoptosis is a prominent feature in neurodegenerative diseases such as AD, PD, amyotrophic lateral sclerosis, and Huntington’s disease, and is associated with multiple cellular processes such as oxidative stress, excitotoxicity, mitochondrial dysfunction, protein misfolding, and inflammation. This article delves into the mechanisms of apoptosis in AD, revealing how Aβ and tau protein deposits trigger apoptotic pathways, including the mitochondrial pathway and the death receptor pathway, leading to neuronal death. Bcl-2 family proteins play a key role in the regulation of mitochondrial membrane permeability and the release of apoptosis factors, while caspase protease, especially Caspase-3, acts as an apoptosis effector molecule to activate downstream apoptosis-related proteins and destroy cytoskeleton and housekeeping gene functions. The paper also highlights the role of oxidative stress and mitochondrial dysfunction in AD, as well as the dual role of p53 protein in DNA repair and apoptosis. In addition, the researchers used network pharmacology and molecular docking techniques to predict the main active ingredients and potential core targets of Chinese medicine compound Jiedu Yizhi formula (JDYZF), and found that JDYZF can inhibit apoptosis by regulating the expression of apoptosis-related genes such as Bcl-2, Bax, and caspase-3. Improving the cognitive function of AD mice provides a new perspective and potential therapeutic strategy for the treatment of AD (Cui T. et al., 2024). In HT22 cell models simulating AD, apoptosis is the main form of neuronal loss, and apoptosis induced by Aβ25-35 fragments is manifested by changes in cell morphology, decreased activity, and increased apoptotic markers. Ganoderic acid A (GAA) protects HT22 cells from AD-related damage by dose-dependent inhibition of MAPK/ERK signaling pathways associated with apoptosis, reducing oxidative stress and mitochondrial dysfunction. In addition, GAA treatment significantly reduced the expression of apoptosis marker caspase-3 and the apoptosis rate, while reducing the expression of AD pathological markers Aβ and p-Tau, revealing the potential neuroprotective effect of GAA in inhibiting the apoptosis of HT22 cells and alleviating the pathological damage of AD (Shao et al., 2024). In addition, apoptosis also plays a role in the development of brain tumors such as glioblastoma, and its defects may lead to the development of brain cancer, and the different morphological and biochemical characteristics of apoptosis pathways are closely related to the regulation of cell death (Lim et al., 2024). In Traumatic Brain Injury (TBI), apoptotic neuronal death serves as a physiological and protective response to injury, enabling the removal of unwanted neurons while minimizing the activation of the immune system (Akamatsu and Hanafy, 2020). However, under pathological conditions, the overactivation of apoptosis-related pathways can lead to detrimental effects. This is exemplified by studies involving transgenic mice that overexpress anti-apoptotic proteins, which demonstrated significant reductions in cortical and hippocampal damage following TBI, highlighting the importance of a balanced apoptotic response in the context of CNS trauma (Haider et al., 2024). As apoptosis is a finely regulated process, oxidative stress ultimately drives apoptosis by impacting molecular and signaling pathways at multiple levels. Therefore, maintaining the balance of intracellular ROS is crucial for preventing apoptosis and preserving cell health (Figure 2).

### 4.2 Necrosis

Oxidative stress-induced cell necrosis, divergent from apoptosis, is a programmed cell death modality involving intricate signaling pathways and molecular mechanisms, with the accumulation of ROS at its core. ROS, stemming from sources such as mitochondrial electron transport chain leakage, NADPH oxidase activation, and inflammatory reactions, initiate a cascade of events leading to cell necrosis. Excessive ROS initially compromise the integrity of the mitochondrial membrane, leading to a decrease in mitochondrial membrane potential and consequently impairing mitochondrial function and energy metabolism. Subsequently, ROS trigger an inflammatory response, marked by an increased release of inflammatory mediators such as tumor necrosis factor α (TNF-α), which can initiate necrosis signaling pathways. Furthermore, elevated ROS levels promote the formation of the necrosome, a complex comprising receptor-interacting protein kinase 1 (RIP1) and RIP3 kinases that phosphorylate mixed lineage kinase domain-like (MLKL) proteins, leading to cell membrane destruction and the release of cellular contents, ultimately resulting in cell necrosis (Chen et al., 2022; Li G. et al., 2024). In the final stages, cell membrane integrity is compromised, causing cellular contents to leak out and trigger inflammation and tissue damage. Under oxidative stress, the p53 protein forms a complex with cyclophilin D (CypD) in the mitochondrial matrix, opening the mitochondrial permeability transition pore and leading to the release of mitochondrial contents, which induces cell necrosis (Fayaz and Rajanikant, 2015). Concurrently, mild oxidative stress can activate the p38/MK2 complex, leading to the phosphorylation of mitochondrial fission factor 1 (MFF1) and the subsequent release of heat shock 60 kDa protein 1 (HSP60), which binds to and activates the inhibitor of kappa B kinase (IKK) complex in the cytoplasm, promoting the expression of Nuclear Factor kappa B (NF-κB)-dependent survival genes in the nucleus and establishing a survival circuit (Wu et al., 2020; Min et al., 2022). Unlike the regulated process of apoptosis, cell necrosis is often an uncontrolled form of cell death associated with various pathological states, such as cerebral ischemia, trauma, infection, and autoimmune diseases. In CNS diseases, necrotic apoptosis is involved in the pathological process of a variety of neurodegenerative diseases, including PD, amyotrophic lateral bundle sclerosis (ALS), multiple sclerosis (MS), and AD (Dominguez et al., 2021; Shi et al., 2023; Huang X.-L. et al., 2024; Zhang T. et al., 2024). Further, necrotic apoptosis may lead to progressive loss and death of nerve cells in TBI(Bao et al., 2019). In addition, necrotic apoptosis inhibitors, such as necrostatin 1 (Nec-1), have received attention for their potential in reducing the production of ROS, suggesting a link between necrotic apoptosis and oxidative stress (Huang et al., 2016). In terms of neuroprotection, a number of small molecules have been found to have neuroprotective effects, which may work by modulating necrotic apoptosis and antioxidant activity (Zhao et al., 2022; Khajepour et al., 2024; Khan et al., 2024). Therefore, the role of necrosis in CNS diseases is closed related to oxidative stress and neuroprotective mechanisms, providing potential targets for the treatment of CNS diseases.

### 4.3 Parthanatos

Oxidative stress-induced parthanatos is a distinct modality of programmed cell death, hallmarked by the overactivation of polyadenosine diphosphoribose polymerase 1 (PARP-1), accumulation of polyadenosine diphosphoribose (PAR) polymers, mitochondrial depolarization, and the nuclear translocation and chromatin condensation of apoptosis-inducing factor (AIF) (Qu et al., 2024). Under oxidative stress, PARP-1 activation leads to the consumption of nicotinamide adenine dinucleotide (NAD+) and adenosine triphosphate (ATP), resulting in cellular energy metabolism dysfunction. This activation induces the release of AIF from mitochondria and its subsequent transfer to the nucleus, causing DNA fragmentation and cell death (Yako et al., 2024). Parthanatos has been implicated in a variety of diseases, including CNS disorders, heart disease, diabetes, and inflammatory diseases, where oxidative stress and PARP-1 activation may contribute to cell death, exacerbating pathological processes (Wang et al., 2023a; Yako et al., 2024; Yang L. et al., 2024; Zhang W. et al., 2024). In CNS diseases, parthanatos is involved in a variety of neuropathological processes, such as oxidative stress, neuroinflammation, mitochondrial dysfunction, excitotoxicity, autophagy damage, and ER stress. These processes are particularly critical in neurodegenerative diseases such as AD, PD, amyotrophic lateral sclerosis and Huntington’s disease (Thapa et al., 2021; Park et al., 2022; 2022). In addition, parthanatos plays a role in the occurrence, progression and treatment of several neurological disorders such as stroke, subarachnoid hemorrhage, multiple sclerosis (MS), epilepsy and neuropathic pain (Wang et al., 2023b; Han et al., 2024; Li C. et al., 2024). An in-depth understanding of the role of parthanatos in the pathological process of these diseases may provide new targets and therapeutic strategies for the treatment of CNS diseases.

### 4.4 Ferroptosis

Oxidative stress-induced ferroptosis is a distinct form of cell death that is contingent upon iron metabolism and characterized by the accumulation of lipid peroxidation to cytotoxic levels. This cell death pathway is initiated by the interplay of ROS, susceptible lipids, and the ensuing lipid peroxidation. Mitochondria, a primary source of ROS, particularly superoxide anions generated during oxidative phosphorylation, plays a pivotal role in the initiation of ferroptosis (Hu et al., 2024). The induction of ferroptosis is predicated on ROS stimulation from diverse sources, including mitochondrial respiration, NADPH oxidase activity, enzymatic reactions, and Fenton chemistry (Endale et al., 2023). Key enzymes in the lipid peroxidation process, such as arachidonic acid-derived lipid oxygenase (ALOX), cyclooxygenase (PTGS), and cytochrome P450, catalyze the peroxidation of lipids, leading to the formation of hydroperoxides. These hydroperoxides can initiate a chain reaction and undergo cleavage reactions, often facilitated by transition metals like iron, resulting in the generation of highly reactive lipid radicals (Trostchansky et al., 2021). System X Cystine Transporter (xCT) transports cystine (Cys) into the cell and glutamate (Glu) out of the cell. Inside the cell, cystine combines with glycine (Gly) to form glutathione (GSH), a process catalyzed by Glutamate Cyste Ligase (GCL) and Glutathione Synthetase (GSS). The antioxidant defense system is crucial in ferroptosis, with Glutathione peroxidase 4 (GPX4) and xCT being the central enzyme that mitigates oxidative stress by reducing lipid hydroperoxides to their corresponding alcohols. The depletion of intracellular GSH, induced by the inhibition of cystine transport proteins, leads to GPX4 inactivation, allowing lipid peroxidation to accumulate to a degree that triggers cell death (Zhang X. et al., 2024; Zhong X. et al., 2024). This mode of cell death, distinct in its molecular underpinnings, is induced by oxidative stress and has implications in a spectrum of diseases.

**FIGURE 3:**
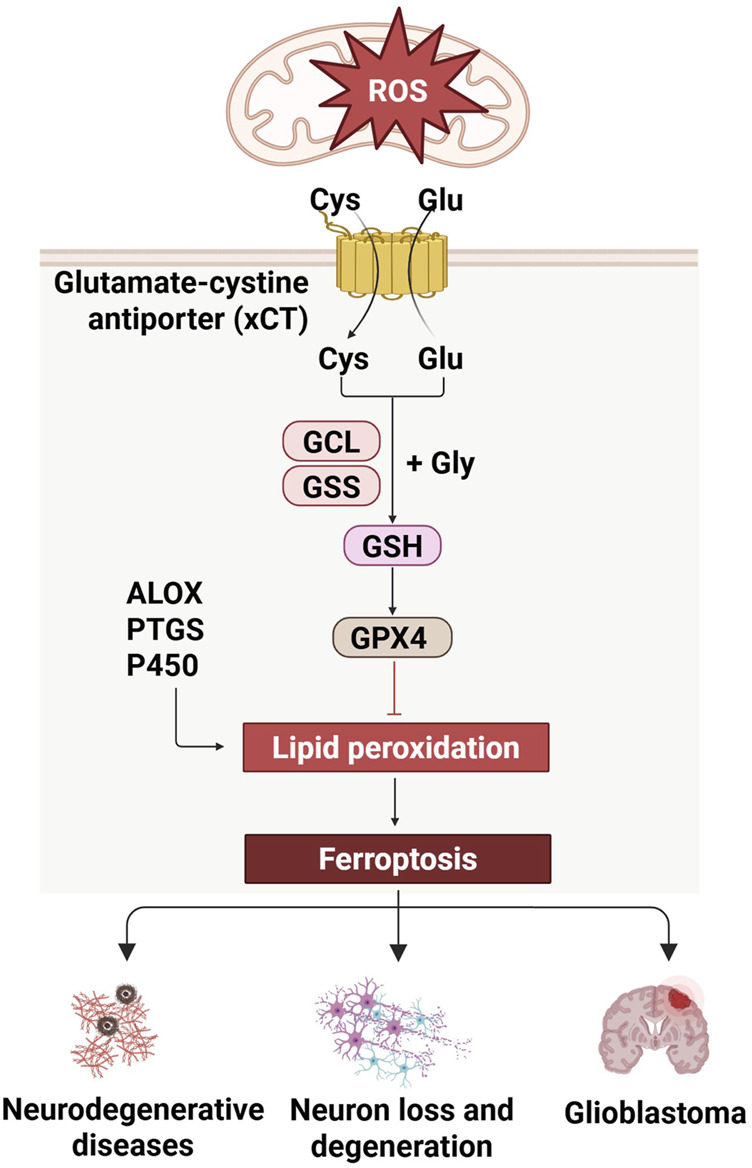
Regulation of ferroptosis in CNS diseases. Ferroptosis, mediated by xCT and GSH pathway, is relevant to CNS diseases, and is linked to neurodegeneration and glioblastoma.

In neurodegenerative diseases, ferroptosis may play a role by affecting oxidative stress in neurons and mitochondrial function. For example, studies have shown that the accumulation of iron in brain samples from people with PD is associated with neuronal ferroptosis, suggesting that ferroptosis may be an important mechanism leading to neuronal loss (Lei et al., 2024; Wang et al., 2024b). In addition, regulatory pathways of ferroptosis, such as the activity of GPX4, are critical for maintaining the REDOX balance of cells, and its dysfunction may lead to ferroptosis in nerve cells (Won et al., 2024). In brain tumors, ferroptosis has also shown potential therapeutic significance. The occurrence, progression and metastasis of brain tumors such as glioblastoma may be related to ferroptosis, and manipulation of iron death may provide a new strategy for the treatment of brain tumors (Chen C. et al., 2024). Nanoparticles serve as multifunctional platforms that can cross the blood-brain barrier and deliver therapeutic drugs to the brain to meet the need for precise visualization of ferroptosis and brain tumor treatment (Minchenko et al., 2024). Overall, the role of ferroptosis in CNS disease involves multiple layers, including the pathological mechanisms of neurodegenerative diseases, the therapeutic potential of brain tumors, and the repair process after CNS damage. The regulation and intervention of ferroptosis may provide new strategies for the treatment of CNS diseases, but the specific mechanisms and applications still need to be further studied and explored (Figure 3).

### 4.5 Pyroptosis

Pyroptosis, a distinct form of programmed cell death, is characterized by cellular swelling leading to membrane rupture, subsequent release of cellular contents, and the initiation of a potent inflammatory response (He X. et al., 2024). This inflammatory cell death pathway is distinct from apoptosis and is mediated by the activation of inflammasomes and the formation of pores in the cell membrane by the gasdermin family of proteins. The canonical pyroptotic signaling pathway is initiated by the recognition of pathogen-associated molecular patterns (PAMPs) and damage-associated molecular patterns (DAMPs) by pattern recognition receptors (PRRs), which assemble the inflammasome complex (Zhao et al., 2024). This assembly results in the activation of procaspase-1 and the subsequent formation of the inflammasome, leading to the cleavage of gasdermin D (GSDMD) and the maturation of interleukin-1β (IL-1β) and IL-18 (Chen D. et al., 2024). The N-terminal domain of GSDMD forms non-selective pores in the cell membrane, causing osmotic lysis and cell death, with IL-1β and IL-18 being released through these pores. Pyroptotic cells exhibit morphological features reminiscent of both necrosis and apoptosis, with the process characterized by the formation of 1–2 nm diameter pores in the cell membrane, potassium ion efflux, and cellular expansion, which trigger an immune response and amplify inflammation. During pyroptosis, the nucleus undergoes rounding, chromatin condensation, and DNA fragmentation, distinct from the morphological changes observed in apoptosis (Wang et al., 2024c). Inflammatory caspases, central to pyroptosis, play a significant role in mediating both apoptosis and inflammation (Bhuiyan et al., 2024). Pyroptosis is implicated in various physiological processes, including cell differentiation, tissue homeostasis, mitochondrial function, immune tolerance, and the formation of neutrophil extracellular traps (NETs).

Recent studies have revealed the complex mechanisms of pyroptosis in the development of CNS disease, including its role in neuroinflammation. Pyroptosis may affect specific brain regions or cell types in different ways, thereby contributing to disease progression in neurodegenerative disease (Bhuiyan et al., 2024; Ghaith et al., 2024). After stroke, the inflammatory response of microglia and swelling of astrocytes are closely related to pyroptosis, and these processes lead to neuronal damage and neuroinflammation (Xu et al., 2024). Therefore, understanding the role of pyroptosis in CNS disease is critical to developing effective treatment strategies.

### 4.6 Paraptosis

Paraptosis is a unique mode of programmed cell death characterized by cytoplasmic vacuolation and damage to the endoplasmic reticulum and mitochondria (Robinson et al., 2024a). Unlike apoptosis and necrosis, paraptosis does not involve the activation of caspase, does not form apoptotic bodies, and does not cause inflammation. During paraptosis, extensive vacuolation occurs in the cytoplasm, but the changes in the nucleus are not obvious, such as nuclear enrichment or nuclear fragmentation, which are typical apoptotic features. In addition, the molecular mechanisms of paraptosis are involved in endoplasmic reticulum stress, unfolded protein response (UPR), ubiquitin-proteasome system (UPS), and changes in autophagy flow (Solovieva et al., 2022). Ion imbalances, ROS, mercapto-disulfide bond equilibria, and REDOX states also play key roles in the initiation and execution of paraptosis (Robinson et al., 2024a). Disruption of any of these components can set off a cascade of reactions that amplify the signal and eventually lead to an irreversible state characterized by significant expansion of the endoplasmic reticulum and mitochondria, manifested as cytoplasmic vacuolation.

In CNS diseases, paraptosis may be associated with a variety of pathological processes, including oxidative stress, neuroinflammation, mitochondrial dysfunction, excitotoxicity, autophagy damage, and ER stress. Paraptosis may play different roles in different CNS diseases. For example, in stroke and neurodegenerative diseases, amyotrophic lateral sclerosis, and Huntington’s disease, paraapoptosis may be involved in the loss and death of neurons (Fricker et al., 2018; Homma et al., 2024; Robinson et al., 2024b). Therefore, the role of paraapoptosis in CNS diseases is multifaceted, involving a variety of molecular mechanisms and pathophysiological processes. A deeper understanding of the molecular mechanisms of paraapoptosis is essential for developing new therapeutic strategies and identifying future research directions.

### 4.7 Oxeiptosis

Oxeiptosis is a newly discovered mode of programmed cell death associated with ROS that is characterized as independent of caspase activation (Ingersoll et al., 2024). This cell death pattern is induced by ROS and is associated with specific molecular mechanisms, specifically the KEAP1/PGAM5/AIFM1 signaling pathway (Guo et al., 2023). Under low levels of oxidative stress, the C-terminal cysteine residue of KEAP1 oxidized, causing it to dissociate from nuclear factor erythroid 2-related factor 2 (NRF2), which then transferred to the nucleus and activated the expression of multiple antioxidant genes. However, at high levels of oxidative stress, KEAP1’s interaction with PGAM5 is lost, causing PGAM5 to transfer into the mitochondria, where PGAM5 dephosphorylates AIFM1, ultimately leading to the occurrence of oxeiptosis. Oxeiptosis plays an important role in the progression of a variety of diseases, including viral infections, inflammatory responses, and tumor suppression (Holze et al., 2018; Oikawa et al., 2022; Wen et al., 2024). For example, the study found that PGAM5^−/−^ mice showed increased inflammatory parameters in an ozone exposure model, suggesting that oxeiptosis plays a negative regulatory role in the inflammatory response when regulating harmful ROS levels. In addition, influenza A virus-mediated inflammation and morbidity increased in PGAM5^−/−^ mice, further supporting the central role of oxeiptosis in preventing overactivation of the immune response during viral infection. The molecular mechanisms and regulatory networks of oxeiptosis have not been fully elucidated, but studies have shown its importance in a variety of biological functions, such as the loss of PGAM5 and AIFM1, which are associated with neurological dysfunction, and KEAP1 mutations, which are associated with lung and prostate cancer (Holze et al., 2018).

In CNS diseases, the role of oxeiptosis is gradually being revealed, especially in Alzheimer’s disease, where the expression of proteins associated with this pathway is upregulated, hinting at its role in oxidative stress and mitochondrial damage. Studies have shown that KEAP1/PGAM5/AIFM1 mediated oxeiptosis may lead to neuron loss in neurodegenerative diseases by causing oxidative stress and mitochondrial dysfunction (Zhong F. et al., 2024). In addition, oxeiptosis has been implicated in neurodevelopment, as the loss of PGAM5 and AIFM1 leads to neurological dysfunction in mice. Therefore, the role of oxeiptosis in CNS diseases is not limited to cell death, but may also involve the regulation of inflammatory responses and neuroprotective mechanisms, providing new potential targets for treatment.

Collectively, oxidative cell death significantly impacts CNS health. It is imperative to explore therapeutic strategies that can effectively mitigate these detrimental effects. One such approach is antioxidant therapy, which aims to restore the balance between ROS production and antioxidant defenses, thereby protecting neurons and other CNS cells from oxidative damage.

## 5 Antioxidant therapy strategies

### 5.1 N-acetylcysteine

The application of N-acetylcysteine (NAC) in the treatment of CNS diseases is multifaceted, with mechanisms of action that include antioxidant, anti-inflammatory, nerve and mitochondrial protection, as well as arterial plaque stabilization and thrombolytic function enhancement. As an antioxidant and free radical scavenger, NAC boosts intracellular GSH at the cellular level, which is crucial for combating neurodegenerative diseases and neuronal death caused by oxidative stress (Vargas-Barona et al., 2024; Zhang et al., 2025). The protective pharmacokinetics of NAC in humans is also discussed. In the context of ischemic stroke, NAC has shown protective effects against ischemic brain injury through mechanisms including anti-oxidation, inhibition of inflammation, protection of cerebral nerve and mitochondrial function, and stabilization of arterial plaque and thrombolytic function (Komakula et al., 2024). Furthermore, NAC mitigated damage to hippocampal neurons after transient global ischemia by reducing matrix metalloproteinase (MMP)-9 activity (Kamel et al., 2024). NAC is capable of restoring cellular glutathione, a key antioxidant that declines with age, and studies have shown that NAC can reduce the risk of brain aging (Kumar et al., 2023). In summary, NAC has broad application prospects in the treatment of CNS diseases, and its antioxidant and neuroprotective effects provide a new strategy for the treatment of various diseases.

### 5.2 Iron chelators

The therapeutic potential of iron chelators in the treatment of CNS diseases is multifaceted, targeting various mechanisms to protect neurons and reduce damage. By regulating iron metabolism in the brain and decreasing the accumulation of iron ions, these agents mitigate oxidative stress and neuroinflammation, which are key contributors to neuronal damage (El Safadi et al., 2024). In the context of Parkinson’s disease, iron chelators have been shown to enhance motor performance and reduce nerve damage. Deferriamine, for instance, as an iron chelating agent, can effectively cross the blood-brain barrier and target dopaminergic neurons through its nanomedical form, specifically clear ROS and iron accumulation in PD focal areas, regulate iron homeostasis and reduce lipid peroxidation, and then inhibit ferroptosis to alleviate the loss of dopaminergic neurons and motor dysfunction (Huang Y. et al., 2024; Lei et al., 2024). In models of AD, amyotrophic lateral sclerosis, and aging, novel multi-target iron chelators have demonstrated neuroprotective effects (Zou et al., 2024). These effects are attributed not only to their iron-chelating properties but also to their anti-apoptotic capabilities. These capabilities contribute to the amelioration of neurodegeneration, enhancement of positive behavioral outcomes, and upregulation of neuroprotective signaling pathways (Zheng et al., 2005). Furthermore, iron chelators such as minocycline have shown the ability to inhibit the activation and proliferation of microglia and matrix metalloproteinases. Through iron chelation, they contribute to neuroprotection by reducing the areas of brain injury following cerebral hemorrhage and improving nerve function deficits (Wang L. et al., 2024). As a novel iron chelating agent and oxidative phosphorylation inhibitor, VLX600 can induce non-caspase-dependent cell death in glioblastoma (GBM) cells (Reisbeck et al., 2023). Collectively, these findings underscore the significant potential of iron chelators in the treatment of CNS diseases, highlighting their dual role in attenuating iron-mediated toxicity and providing neuroprotection *via* multiple signaling pathways.

### 5.3 Activator of antioxidant enzymes

The utilization of antioxidant activators in the treatment of CNS diseases is multifaceted, targeting the modulation of key signaling pathways to enhance cellular antioxidant defenses. Initially, the NRF2 signaling pathway is crucial for the cellular response to oxidative stress. A decline in NRF2 expression with age leads to an imbalance in the oxidative stress response, suggesting that the NRF2 pathway is a promising target for neurodegenerative disease therapy (Qin et al., 2024). NRF2 activators induce the expression of antioxidant enzymes, including SOD, catalase (CAT), and GPX, which are pivotal in mitigating oxidative damage (Luo et al., 2024). The activation of the Keap1-Nrf2-ARE signaling pathway in neurodegenerative disease models has been shown to provide cellular antioxidant protection, reduce neuronal damage, and delay disease progression (Chakkittukandiyil et al., 2024). Furthermore, the PI3K/Akt signaling pathway plays a significant role in the protective effects against ischemic stroke. By inhibiting oxidative stress, inflammatory responses, and matrix metalloproteinases expression, this pathway can ameliorate BBB damage in ischemic stroke, offering a novel therapeutic strategy. Additionally, the contribution of amyloid-beta (Aβ), Tau, and α-synuclein to BBB damage in neurodegenerative diseases is substantial. These pathological proteins can directly affect BBB integrity by influencing key BBB components such as pericytes and endothelial cells, or indirectly by promoting brain macrophage activation and dysfunction (Li et al., 2025; Wu et al., 2025). In summary, antioxidant activators exert their therapeutic effects by activating the Nrf2 signaling pathway to upregulate antioxidant enzyme expression, inhibiting oxidative stress and inflammation through the PI3K/Akt signaling pathway, and reducing BBB damage. These mechanisms highlight the significant potential and application prospects of antioxidant activators in the treatment of CNS diseases, providing a foundation for developing novel therapeutic strategies to combat neurodegenerative conditions.

### 5.4 Other strategies

**FIGURE 4:**
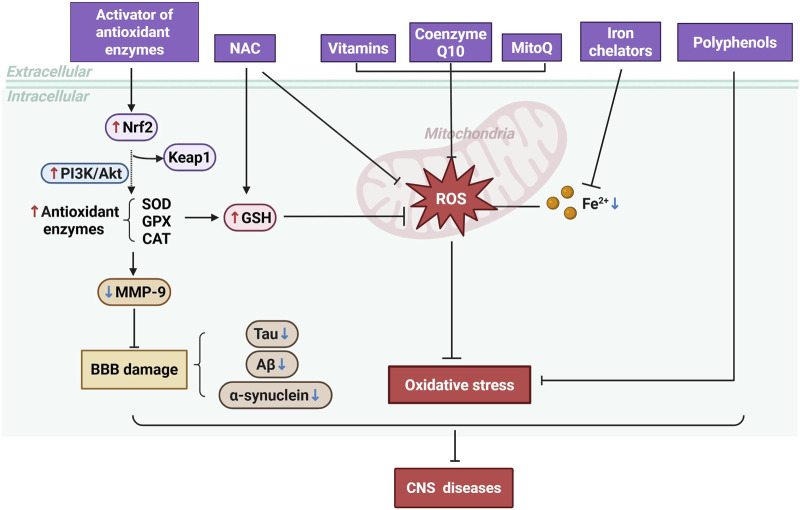
Mechanisms of Antioxidant Therapy in CNS Diseases. Antioxidant therapies, including the activation of antioxidant enzymes, administration of NAC, vitamins, Coenzyme Q10, MitoQ, iron chelators, and polyphenols, work to reduce ROS levels.

Vitamins C and E, along with Coenzyme Q10, directly scavenge reactive oxygen species, reducing oxidative damage to cellular components (Fuchs et al., 2023). Mitochondrial-targeted antioxidants like MitoQ effectively reduce oxidative stress by directly targeting mitochondria, which are key players in neurodegenerative diseases (Ibrahim et al., 2023). Furthermore, polyphenols including curcumin and small molecules have shown neuroprotective effects by modulating various cellular signaling pathways involved in oxidative stress and inflammation, and their ability to protect neuronal function makes them promising candidates for further study in neurodegenerative diseases (Alecu et al., 2025; Awad et al., 2025) (Figure 4).

### 5.5 Challenges and prospects of antioxidant therapy

Antioxidant therapy in the context of CNS diseases is confronted with numerous challenges, such as the high incidence of failed clinical trials, the intricate role of oxidative stress in disease pathology, the impediment posed by the blood-brain barrier to drug permeation, and the specific needs for activators of antioxidant enzymes. NAC has shown neuroprotective effects in animal models and some clinical trials for ischemic stroke and neurodegenerative diseases, reducing oxidative stress and neuronal damage. However, while antioxidants are generally considered safe, they can have side effects. For instance, long-term use of NAC may cause some gastrointestinal reactions, and high doses of vitamin E have been associated with an increased risk of hemorrhagic stroke (Le et al., 2020; Schwalfenberg, 2021). Minocycline, an iron chelator, reduces microglial activation but can cause dizziness (McKean et al., 2024; Zhou et al., 2024). Long-term outcomes are still under investigation. Despite these obstacles, the future of antioxidant therapy remains auspicious. An enhanced comprehension of oxidative stress mechanisms, coupled with the emergence of innovative therapeutic strategies—including the development of antioxidant enzyme activators, the utilization of nanotechnology to facilitate drug delivery, and the modulation of Nrf2 and PI3K/Akt signaling pathways—offers novel strategies and methodologies for addressing CNS diseases. Experimental models, both *in vitro* and *in vivo*, are essential for studying CNS diseases and evaluating treatments. *In vitro* models offer controlled, quick, and cost-effective studies, facilitating detailed analysis but lack the complexity of *in vivo* environments. *In vivo* models better mimic human diseases, including immune responses, yet they can show species-specific responses and ethical concerns. Enhancing model accuracy with humanized mice or advanced imaging can improve clinical applicability. Additionally, emerging therapies and technologies, such as real-time *in vivo* imaging of ROS in the CNS and the application of CRISPR/Cas9 technology, offer promising avenues for more precise, targeted, and personalized treatments. Nevertheless, these novel approaches necessitate further development and careful consideration of their potential risks and benefits. In summary, while antioxidant therapy holds promise for the treatment of CNS diseases, additional research is essential to address current gaps and optimize clinical outcomes (Table 1).

**TABLE 1:** Overview of antioxidant therapy Strategies for central nervous system diseases. This table compares antioxidant therapies for CNS diseases, noting their mechanisms, benefits, limitations, and clinical efficacy.

Antioxidant Therapy	Mechanism of Action	Advantages	Limitations	Model	Clinical Efficacy	References
NAC	Increase GSH, scavenge free radicals, reduce oxidative stress	Broad-spectrum	Gastrointestinal issues	Animal Human	Protective effects in ischemic brain injury and neurodegenerative diseases	Vargas-Barona et al. (2024), Zhang et al. (2025), Komakula et al. (2024), Kamel et al. (2024), Kumar et al. (2023), Schwalfenberg (2021)
Iron Chelators	Reduce iron accumulation, decrease oxidative stress	Effective in reducing iron overload	Side effects like gastrointestinal issues, dizziness, limited blood-brain barrier penetration	Animal Human	Improve motor performance in Parkinson’s disease	(El Safadi et al., 2024, (Huang et al., 2024d; Lei et al., 2024; Zou et al., 2024; Zheng et al., 2005; Wang et al., 2024a; Reisbeck et al., 2023; McKean et al., 2024; Zhou et al., 2024)
Antioxidant Enzyme Activators	Activate Nrf2 pathway, upregulates SOD, GPX, CAT	Potential for long-term neuroprotection	Limited bioavailability, potential for off-target effects	Animal	Promising results in preclinical models, Limited clinical data available	Qin et al. (2024), Luo et al. (2024), Chakkittukandiyil et al. (2024), Li et al. (2025), Wu et al. (2025)
Vitamins and Coenzyme Q10	Directly scavenge ROS	Natural and well-tolerated, Synergistic effects with other antioxidants	Limited efficacy as monotherapy, Variable bioavailability	Animal Cell Human	Potential benefits in combination therapies	Fuchs et al. (2023)
Mitochondrial-Targeted Antioxidants	Reduce mitochondrial oxidative damage	High specificity for mitochondria	Limited clinical data, potential for mitochondrial toxicity	Animal	Protective effects in preclinical models. Limited clinical trials	Ibrahim et al. (2023)
Polyphenols and Small Molecules (e.g., Curcumin)	Modulate cellular signaling pathways, reduce inflammation, scavenge ROS	Potential for crossing the blood-brain barrier	Poor bioavailability, limited long-term stability	Animal Human	Mixed results in clinical trials, potential for neuroprotection in combination therapies	Alecu et al. (2025), Awad et al. (2025)

## 6 Conclusion

Oxidative cell death plays a central role in CNS diseases, and addressing its multifaceted nature is crucial for disease management. Despite advancements in our understanding of oxidative cell death in the CNS, significant gaps remain. For instance, the precise mechanisms of oxidative cell death initiation and propagation in CNS diseases are not fully elucidated, and the roles of ROS in interacting with cellular signaling pathways are not completely understood. Additionally, the lack of effective biomarkers for early detection and monitoring of oxidative stress and cell death in CNS diseases hinders diagnosis and treatment monitoring. Personalized medicine, by considering genetic variations affecting antioxidant enzyme function or stress response pathways, could enhance oxidative stress therapies and potentially improve treatment outcomes. More clinical trials are necessary to verify the efficacy and safety of antioxidants in human patients, particularly in the early stages of disease. Future research should focus on elucidating the molecular mechanisms underlying oxidative cell death in the CNS and developing novel therapeutic agents targeting both oxidative stress and inflammation. Advanced techniques such as single-cell sequencing, real-time *in vivo* imaging of ROS, and CRISPR/Cas9-based genetic editing could provide valuable insights into these mechanisms and lead to more effective treatments.
